# Two Permeases Associated with the Multifunctional CtaP Cysteine Transport System in Listeria monocytogenes Play Distinct Roles in Pathogenesis

**DOI:** 10.1128/spectrum.03317-22

**Published:** 2023-05-18

**Authors:** Diandra M. Vaval Taylor, Bobbi Xayarath, Nancy E. Freitag

**Affiliations:** a Department of Pharmaceutical Sciences, University of Illinois at Chicago, Chicago, Illinois, USA; University of North Dakota

**Keywords:** CtaP, ABC transporters, cysteine transport, PrfA, bacterial adhesion, virulence, virulence factors

## Abstract

The soil-dwelling bacterium Listeria monocytogenes survives a multitude of conditions when residing in the outside environment and as a pathogen within host cells. Key to survival within the infected mammalian host is the expression of bacterial gene products necessary for nutrient acquisition. Similar to many bacteria, L. monocytogenes uses peptide import to acquire amino acids. Peptide transport systems play an important role in nutrient uptake as well as in additional functions that include bacterial quorum sensing and signal transduction, recycling of peptidoglycan fragments, adherence to eukaryotic cells, and alterations in antibiotic susceptibility. It has been previously described that CtaP, encoded by *lmo0135*, is a multifunctional protein associated with activities that include cysteine transport, resistance to acid, membrane integrity, and bacterial adherence to host cells. *ctaP* is located next to two genes predicted to encode membrane-bound permeases *lmo0136* and *lmo0137*, termed CtpP1 and CtpP2, respectively. Here, we show that CtpP1 and CtpP2 are required for bacterial growth in the presence of low concentrations of cysteine and for virulence in mouse infection models. Taken together, the data identify distinct nonoverlapping roles for two related permeases that are important for the growth and survival of L. monocytogenes within host cells.

**IMPORTANCE** Bacterial peptide transport systems are important for nutrient uptake and may additionally function in a variety of other roles, including bacterial communication, signal transduction, and bacterial adherence to eukaryotic cells. Peptide transport systems often consist of a substrate-binding protein associated with a membrane-spanning permease. The environmental bacterial pathogen Listeria monocytogenes uses the substrate-binding protein CtaP not only for cysteine transport but also for resistance to acid, maintenance of membrane integrity, and bacterial adherence to host cells. In this study, we demonstrate complementary yet distinct functional roles for two membrane permeases, CtpP1 and CtpP2, that are encoded by genes linked to *ctaP* and that contribute to bacterial growth, invasion, and pathogenicity.

## INTRODUCTION

Listeria monocytogenes is a Gram-positive bacterium that survives as a saprophyte in soil while maintaining the capacity to invade and multiply within mammalian cells (1, 2). This non-spore-forming bacterium survives in the outside environment largely as a result of its ability to withstand a myriad of environmental conditions that include large fluctuations in pH, salt concentration, and temperature (3–5). L. monocytogenes can additionally tolerate and adapt to long periods of nutrient starvation without any detectable loss of bacterial virulence (6). This physiological adaptability contributes to the capacity of L. monocytogenes to contaminate food products and replicate despite common methods used for preservation. As a result, L. monocytogenes has been responsible for some of the deadliest foodborne outbreaks and most expensive food recalls in U.S. history (2, 7, 8). Following its ingestion by susceptible individuals, L. monocytogenes passes through the stomach and translocates across the gastrointestinal barrier. The bacterium then disseminates and replicates initially within the liver and spleen with subsequent spread to the central nervous system in immunocompromised adults or to placental and fetal tissue in pregnant women (9–11).

While L. monocytogenes appears to exhibit considerable flexibility with respect to stressful growth conditions, the bacterium retains less flexible but essential nutritional requirements for growth both inside and outside host cells. L. monocytogenes in broth culture requires the presence of several amino acids and vitamins, including leucine, isoleucine, valine, cysteine, methionine, riboflavin, thiamine, and biotin (12, 13). The requirements for growth of the bacterium within the mammalian cytosol continue to be defined but include the preferred use of glycerol or other three-carbon sugars, lipoic acid, and peptides (14, 15).

To fulfill its nutritional requirements, L. monocytogenes has a large number of genes that encode products that have both demonstrated and predicted transport functions. Many gene products are for the acquisition of carbohydrates as part of the phosphoenolpyruvate-dependent phosphotransferase (PTS) system, of which many appear important for acquisition of environmental carbon sources (16, 17). While bacterial replication within the cytosol relies on alternative carbon utilization pathways such as those involved in the uptake of phosphorylated sugars (hexose phosphates) and glycerol and the scavenging of lipoic acid and branched-chain fatty acids (15, 18), experimental evidence indicates that at least some PTS-associated transport systems are required for cytosolic growth (19–21). Two distinct oligopeptide transport systems have been described in L. monocytogenes for the acquisition of host-derived peptides, which are the preferred sources of amino acids (22); these are the Dtp and OppA systems (23–26). Dtp is associated with the transport of dipeptides and tripeptides (26), while the Opp transport system has been associated with the import of peptides ranging from 4 to 35 amino acids in length (27, 28). In addition to peptide import as a source of amino acids, peptide transport systems have been associated with a variety of other functions that include bacterial quorum sensing and signal transduction, recycling of peptidoglycan fragments, adherence to eukaryotic cells, alterations in antibiotic susceptibility, survival under stress conditions, hemolytic activity, and modulation of the activity of the central virulence regulator, PrfA (29–39).

In addition to the Dtp and OppA peptide transport systems, we have previously described the identification and characterization of CtaP, a secreted substrate-binding protein associated with a multifunctional ABC transport system in L. monocytogenes (40). *ctaP*, originally designated *lmo0135*, was annotated as encoding an oligopeptide-binding protein; however, we found that the protein is associated with high-affinity cysteine transport. CtaP facilitates growth in the presence of low levels of cysteine in broth culture (41). It is not required for bacterial replication within the cytosol, where cysteine is apparently not limiting, but does have additional roles associated with bacterial virulence, such as functioning as a cell adhesion molecule and contributing to acid resistance and membrane integrity. In addition, CtaP appears to contribute to the uptake of the pPplA peptide pheromone, which contributes to both bacterial aggregation in broth culture and bacterial escape from host cell vacuoles during cellular infection (41). *ctaP* is adjacent to *lmo0136* and *lmo0137*, genes that are predicted to encode two membrane-bound permease proteins whose functions have not yet been characterized. Here, we examined the roles of the gene products encoded by *lmo0136* and *lmo0137* and provide evidence that the two permeases play distinct yet functionally related roles in association with CtaP by contributing to bacterial growth in the presence of low concentrations of cysteine and to bacterial virulence in mouse infection models.

## RESULTS

### Organization of the CtaP-associated permease gene cluster.

**FIG 1:**
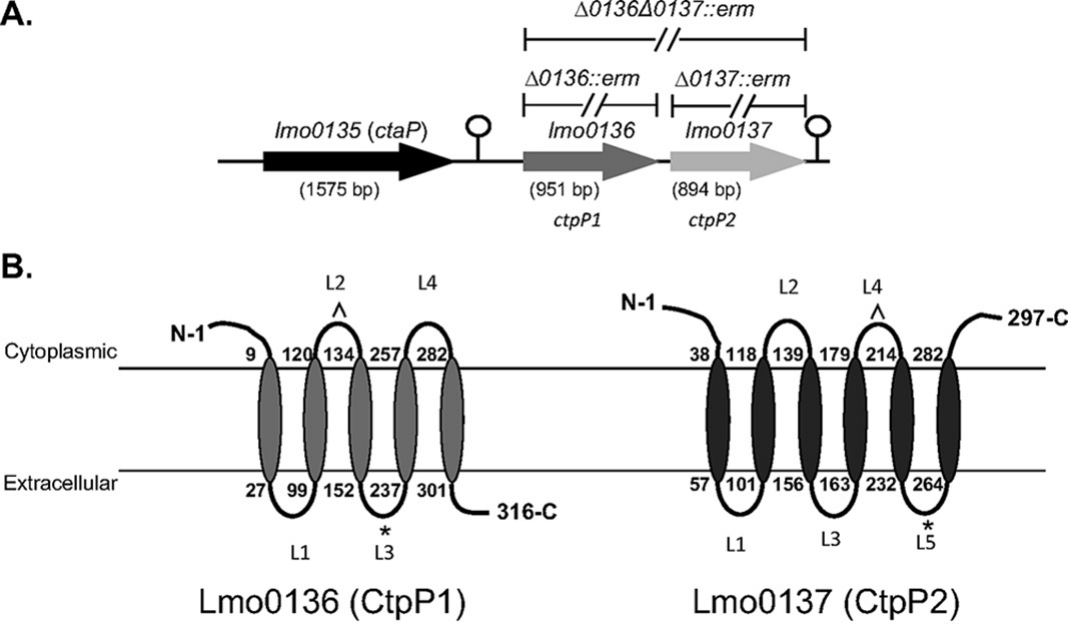
Location and topology of the CtaP permeases. (A) Organization of the CtaP permeases. Dashed lines indicate locations of the specific deletion mutations constructed. (B) Predicted topology of Lmo0136 and Lmo0137. ^, predicted ATP binding loop; *, predicted to interact with the substrate-binding protein CtaP.

Genes encoding ABC transport systems are often found clustered together, such that the gene encoding the substrate-binding protein is located adjacent to the gene encoding the membrane permease (42–45). CtaP functions as a substrate-binding protein, and *ctaP* is located immediately upstream of two genes (*lmo0136* and *lmo0137*) whose products are both predicted to be membrane-associated permease proteins (Fig. 1A). Lmo0136 and Lmo0137 are predicted to be integral transmembrane proteins that could potentially function together to form a channel in the bacterial cell membrane to facilitate substrate transport; alternatively, the two permeases could function as separate and distinct channels. Based on shared homology with other known permeases, an Lmo0136 permease loop (L2) located on the cytoplasmic face of the membrane binds ATP, while another loop (L3) on the extracellular side of the membrane is predicted to interact with the substrate-binding protein CtaP (Fig. 1B). Lmo0136 shares 51% amino acid similarity with Lmo0137, with the highest degree similarity occurring between the predicted ATP-binding domains (L2 of Lmo0136 and L4 of Lmo0137) and the substrate-binding protein-binding domains L3 of Lmo0136 and L5 of Lmo0137 (Fig. 1B). Lmo0136 and Lmo0137 share 55% and 61% amino acid sequence similarity to Lmo2195 and Lmo2194, respectively, the permeases associated with the previously described OppA ABC-dependent transport system in L. monocytogenes. Based on their proximity to *ctaP* and shared sequence homology with other permeases, we have designated Lmo0136 and Lmo0137 CtpP1 and CtpP2, for cysteine transport permease protein 1 and protein 2, respectively.

### CtpP1 and CtpP2 are required for optimal bacterial growth in broth culture.

The substrate-binding protein CtaP has previously been demonstrated to contribute to bacterial growth in rich broth media (40). We therefore generated in-frame deletion mutants of each permease gene and a double deletion mutant of both genes and examined growth of the mutant strains in broth culture. To facilitate the construction of the deletion mutants, the coding regions of *ctpP1*, *ctpP2*, or both *ctpP1* and *ctpP2* were deleted and replaced with the *erm*(B) gene encoding resistance to erythromycin (Fig. 1A). As had been previously found during the construction of the *ctaP* deletion mutant, it was necessary to use drug selection for mutant isolation, as all mutants were found to exhibit growth defects (see below) that prevented their isolation in the absence of antibiotic selection.

**FIG 2:**
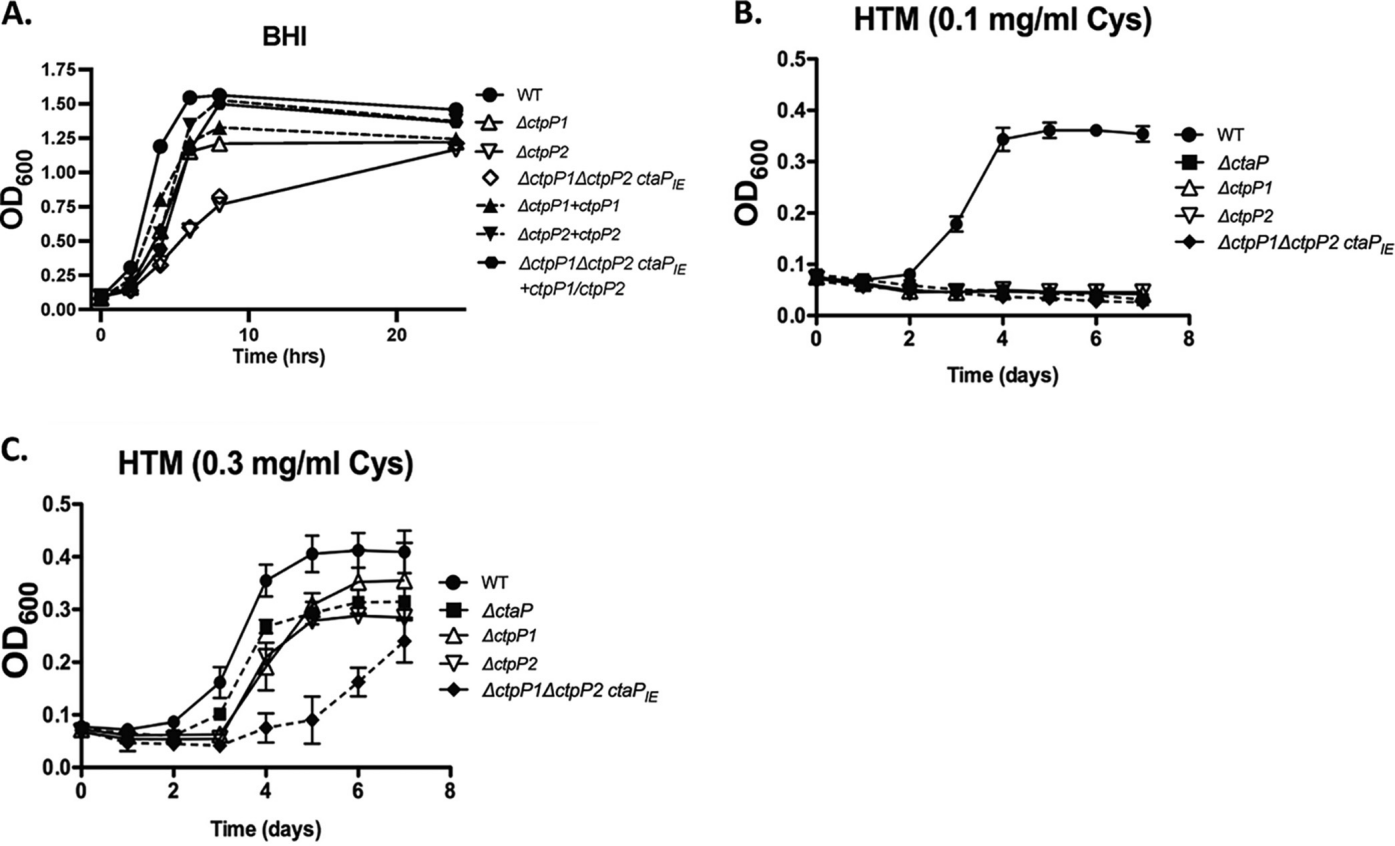
*ctpP1* and *ctpP2* exhibit different growth patterns in broth culture. (A) Assessment of growth of the *ctaP* transporter, permease mutants, and complements in BHI broth culture media. Overnight cultures of each strain grown shaking in BHI at 37°C were diluted 1:20 in fresh BHI media, and the OD_600_ was measured at the indicated time points. (B and C) Assessment of growth of the *ctaP* transporter, permease mutants, and complements in defined culture media. Cultures were grown overnight shaking in BHI at 37°C. Strains were normalized by the OD_600_, pelleted, and washed 2 times in PBS before resuspension in PBS to a final volume equal to the normalized culture volume. Bacterial suspensions were diluted 1:20 in HTM media containing the indicated concentrations of free cysteine. Growth was measured as the OD_600_ at indicated time points.

**FIG 3:**
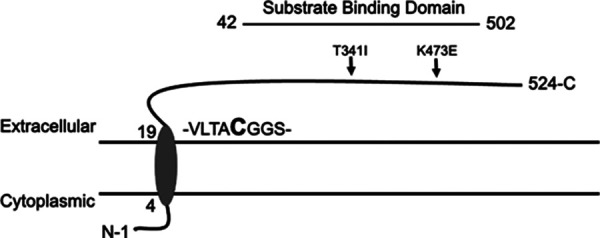
Mutations in the *ctaP* gene of the Δ*ctpP1* Δ*ctpP2* double deletion mutant shown in the L. monocytogenes reference strain 10403S. CtaP protein-coding region with predicted protein domains. Arrows indicate amino acid changes identified in CtaP’s substrate-binding region due to single-nucleotide polymorphisms at residues T341 and K473.

Similar to the Δ*ctaP* mutant, the Δ*ctpP2* and Δ*ctpP1* Δ*ctpP2* double deletion exhibited small-colony phenotypes when grown on BHI agar (data not shown). In contrast, the Δ*ctpP1* deletion mutant exhibited an intermediate colony size that was larger than that of the Δ*ctaP* or Δ*ctpP2* mutants but smaller than that of the wild-type strain. A comparable pattern of growth was observed for strains grown in brain heart infusion (BHI) and chemically defined medium (CDM) broth culture (Fig. 2A; see also Fig. S1 in the supplemental material). Of note, despite the absence of both permeases, the Δ*ctpP1* Δ*ctpP2* double mutant phenotype was identical to that of the single Δ*ctpP2* permease mutant, initially suggesting that *ctpP2* is the predominant functional permease under these conditions. However, when the permease mutants were complemented, the delayed growth patterns of the mutants were rescued, with the exception of the double deletion mutant (Fig. 2B; Fig. S1). Based on the complete failure of efforts to complement the double deletion mutant, both the single and double deletion strains were subjected to whole-genome DNA sequencing. Sequencing revealed that the strain containing the deletion of both *ctpP1* and *ctpP2* acquired two additional mutations, both within *ctaP*: a threonine-to-isoleucine substitution at position 341 (T341I), and a lysine-to-glutamate substitution at position 473 (K473E) (Fig. 3). Both substitutions lie within the predicted extracellular substrate-binding domain of CtaP. Given the nature of the substitutions, the fact that two distinct mutations occurred within *ctaP*, and the failure of wild-type copies of *ctpP1* and *ctpP2* to complement the double deletion strain while the single mutants exhibited at least partial complementation, we speculate that these two amino acid substitutions inactivate CtaP. Loss of CtaP activity based on the amino acid substitutions remains to be directly demonstrated, and it is alternatively possible that the mutations serve to alter CtaP activity rather than completely inactivate the protein. Based on the difficulty of isolation of the original double permease deletion mutant, it would appear that selective pressure exists that leads to mutations in *ctaP* when both functional permeases are missing. While the Δ*ctpP1* Δ*ctpP2* double deletion strain thus also contains a mutant form of *ctaP*, we decided to continue to include the mutant in subsequent assays to observe the consequences of the triple mutation. Hence, we refer to the double deletion permease genes plus mutant *ctaP* as Δ*ctpP1* Δ*ctpP2 ctaP*_IE_ (IE for the new amino acid changes).

### CtpP1 and CtpP2 are required for growth under conditions of limiting cysteine.

CtaP, the substrate-binding component associated with CtpP1 and CtpP2, is required for L. monocytogenes high-affinity transport of cysteine in Hsiang-Ning Tsai medium (HTM). Wild-type L. monocytogenes 10403S is auxotrophic for cysteine and methionine, and growth occurs when these two amino acids are provided at a concentration of 0.1 mg/mL. The Δ*ctaP* mutant is unable to grow in HTM containing these low concentrations of cysteine and methionine, but the growth can be rescued by the addition of increased amounts of free cysteine (0.3 mg/mL), presumably due to the uptake of the amino acid via an alternate and/or low-affinity transport system. To determine whether the permease mutants display a similar requirement for high concentrations of cysteine, the single and double permease deletion mutants were assessed for their ability to grow in HTM containing either low (0.1 mg/mL) or a high (0.3 mg/mL) cysteine in the presence of 0.1 mg/mL methionine. All of the permease deletion mutants (Δ*ctpP1*, Δ*ctpP2*, and Δ*ctpP1* Δ*ctpP2 ctaP*_IE_) failed to grow in minimal media containing low concentrations of cysteine. Increasing the concentration of free cysteine rescued growth of all the mutants but to various degrees (Fig. 2C). The Δ*ctpP1* and Δ*ctpP2* single mutants exhibited patterns of growth that were similar to that of the Δ*ctaP* mutant. Although the Δ*ctpP1* Δ*ctpP2 ctaP*_IE_ mutant exhibited growth in rich BHI media that was comparable to that of the Δ*ctaP* or Δ*ctpP2* single deletion strains, growth of the Δ*ctpP1* Δ*ctpP2 ctaP*_IE_ mutant was only partially restored in HTM supplemented with 0.3 mg/mL cysteine (Fig. 2C). Bacterial culture densities eventually reached those observed for the single mutants in the presence of high cysteine; these cultures were found to often give rise to faster-growing colony variants, suggesting the acquisition of a suppressor mutation(s) (data not shown). Taken together, these results indicate that, similar to CtaP, the permease proteins CtpP1 and CtpP2 are required for bacterial growth in the presence of low concentrations of cysteine.

### CtpP1 and CtpP2 are not required for intracellular replication but contribute to host cell invasion.

**FIG 4:**
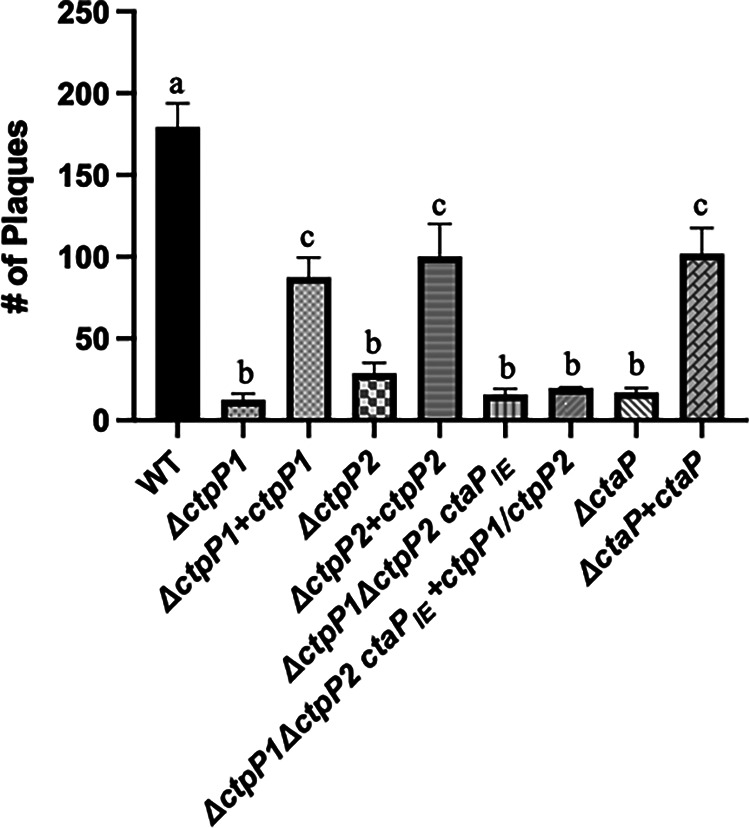
*ctpP1* and *ctpP2* contribute to host cell invasion, based here on measurement of cell-to-cell spread in murine L2 fibroblasts infected with an MOI of 10:1. Plaques were counted 3 days postinfection, and the number of plaques (± the standard error of the mean) were plotted. Data shown are representative of three independent experiments. Statistical significance is indicated with letters b or c comparing WT versus the indicated mutant strains (Mann-Whitney two-tailed nonparametric test for comparison): a, nonsignificantly different from WT; b, *P* < 0.0001; c, *P* < 0.005.

L. monocytogenes is known to preferentially use peptides versus free amino acids during growth in the cytosol of infected host cells (22). We therefore examined if, similar to the Δ*ctaP* mutant, normal patterns of bacterial growth would be observed within the cytosol of infected host cells. The ability of the permease mutants to infect, escape the phagosome, replicate, and spread to adjacent host cells was measured based on the ability of the mutant strains to form visible zones of cell clearing (or plaques) in monolayers of mouse L2 fibroblast cells (46). The number of plaques formed relative to bacterial CFU by the mutants were approximately 5- to 6-fold lower than the number of plaques formed by the wild-type strain (Fig. 4), while the plaques sizes were the same (data not shown), indicating that once mutants were successful at gaining entry into host cells, normal intracellular growth and spread could occur. The overall reduction in the number of plaques formed by the permease mutants was similar to the reduction seen in the number of plaques formed by the Δ*ctaP* mutant in L2 cells, suggesting there is a possible defect with host cell adhesion. Each of the single permease deletions could be partially complemented for plaque formation by the introduction of a wild-type copy of the gene (Fig. 4). CtaP has been demonstrated to directly mediate host cell adhesion (40); therefore, it is possible that the defects observed in the permease mutants with regard to host cell adhesion reflect defects in CtaP binding. Alternatively, both peptide- and sugar-binding permeases of known and unknown substrate specificity have been described to mediate host cell adherence in other bacterial species (36, 47–48).

### CtpP1 and CtpP2 contribute to virulence *in vivo*.

**FIG 5:**
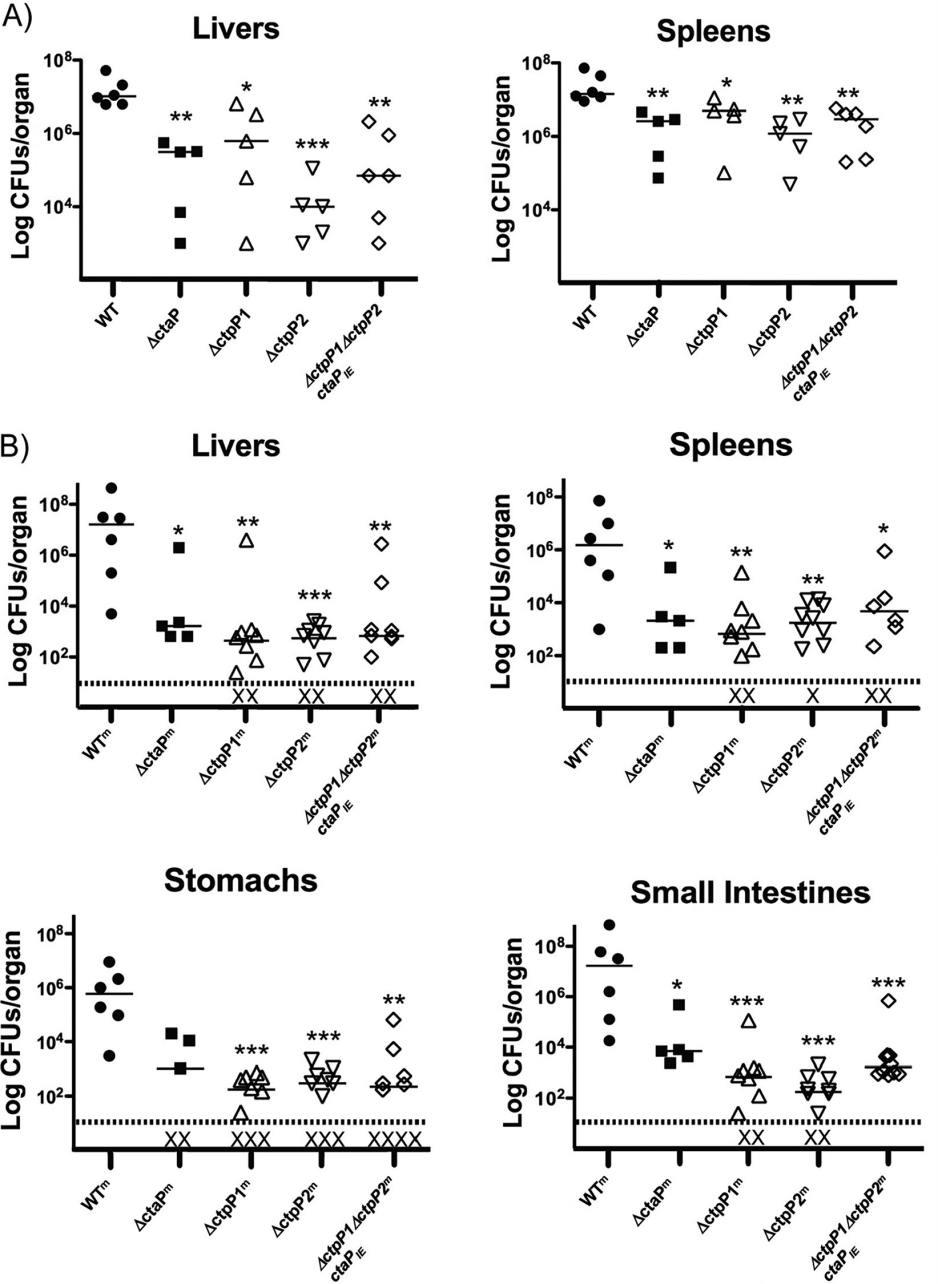
CtpP1 and CtpP2 contribute to virulence *in vivo*. (A) Eight- to 10-week-old female Swiss Webster mice were intravenously infected with 2 × 10^4^ CFU of the wild type, Δ*ctaP*::*erm*, or permease mutant strains. Target organs were harvested 72 h postinfection for the determination of bacterial burdens. (B) Intragastric inoculation of 8- to 10-week-old C57BL/6 mice with 1 × 10^8^ CFU of either L. monocytogenes *inlA^m^*, *inlA^m^* Δ*ctaP*::*erm*, or *inlA^m^* containing the various permease deletions. Statistical significance is indicated for WT versus indicated mutant strain comparisons, using Mann-Whitney two-tailed nonparametric test: *, *P* < 0.05; **, *P* < 0.01; ***, *P* < 0.001.

Δ*ctaP* mutants are significantly attenuated for virulence in mouse models of infection, following either intravenous and intragastric routes of inoculation. Our data thus far suggest, based on similar mutant phenotypes, that the two permease proteins may function in association with CtaP, and thus mutants lacking the permeases would also be anticipated to have decreased virulence. The single and double permease mutants were thus examined in both intravenous and intragastric models of infection. Groups of 8- to 10-week-old female mice were inoculated either through the tail vein with 2 × 10^4^ CFU of the wild-type, Δ*ctaP*::*erm*, or permease mutant strains, or via intragastric inoculation with 1 × 10^8^ CFU of wild-type, Δ*ctaP*::*erm*, or the permease mutant strains, all of which contained the murinized *inlA^m^* allele to enhance intragastric infection (49). Target organs were harvested 72 h postinfection for the determination of bacterial burdens. Following intravenous inoculation, the number of bacterial CFU recovered from both the livers and spleens of mice infected with Δ*ctpP2* or Δ*ctpP1* Δ*ctpP2 ctaP*_IE_ were approximately 1 to 2 logs lower than the number recovered from animals infected with the wild-type strain, while the bacterial burdens recovered from the Δ*ctpP1* mutant were approximately 5-fold lower (Fig. 5A). Intragastric inoculation of groups of 8- to 10-week-old C57BL/6 mice with 1 × 10^8^ CFU of either L. monocytogenes *inlA^m^*, *inlA^m^* Δ*ctaP*::*erm*, or *inlA^m^* containing the various permease deletions revealed that all of the mutants examined were severely attenuated for virulence. The number of bacteria recovered from the organs of mice infected with any of mutant strains were at least 3 to 4 logs lower than the numbers recovered from the organs of mice infected with L. monocytogenes *inlA^m^* (Fig. 5B). The significant reductions in bacteria recovered from the stomachs and intestines of animals infected with the mutant strains suggest a critical role for CtaP and its associated permeases in gastric survival and intestinal colonization.

## DISCUSSION

L. monocytogenes is notable for its capacity to survive and replicate in a myriad of environments both inside and outside mammalian hosts. Secreted gene products have been associated with multiple and sometimes seemingly diverse functions that promote bacterial survival. CtaP was first identified for its roles in the acquisition of cysteine, contributions to surface hydrophobicity, and cell adhesion (40). Here, we provide evidence that the two genes adjoining *ctaP*, *ctpP1* and *ctpP2*, encode apparent permeases that similarly contribute to cysteine uptake, bacterial invasion, and virulence. The adaptation of a transport complex for multiple functional roles may represent one strategy by which L. monocytogenes maintains virulence factors as a saprophyte in the absence of contact with mammalian hosts.

CtaP and its associated permeases appear to function as part of a high-affinity cysteine transport system that promotes growth in the presence of low levels of free cysteine. This uptake system used by L. monocytogenes for cysteine and cystine import under low nutritional conditions thus joins the ABC transporter TcyKLMN, which was recently reported as another cysteine transport system (50). In addition, Loh et al. reported the identification of seven *S*-adenosylmethionine riboswitches located upstream of genes encoding proteins predicted to function in methionine or cysteine metabolism and/or transport, one of which is predicted to be located immediately upstream of *ctaP*, thus lending further support to its role in cysteine acquisition and metabolism (51). Similar to the Δ*ctaP* mutants, L. monocytogenes strains missing one or both CtpP1/CtpP2 permeases (plus the *ctaP* mutation, i.e., Δ*ctpP1* Δ*ctpP2 ctaP*_IE_) exhibited severe growth defects in BHI media, CDM, and in defined medium containing low concentrations of cysteine. The double deletion of the permeases Δ*ctpP1*Δ*ctpP2* resulted in two single-nucleotide polymorphisms occurring within the coding region of *ctaP*, suggesting that one of the permeases must be present in the presence of a functional *ctaP*.

As a general observation, the loss of CtpP1 resulted in phenotypes that were less severe than those of mutants lacking either CtaP or CtpP2, suggesting that these latter proteins have greater physiological roles under the conditions examined. It is possible that there is a higher-affinity association between CtaP and CtpP2 than between CtaP and CtpP1. The phenotypes of the individual permease mutants indicate, however, that CtpP2 does not fully compensate for the loss of CtpP1, indicating that each permease makes distinct contributions to L. monocytogenes physiology.

Each permease appears to be required for efficient bacterial uptake and entry into host cells, as indicated by the decreased number of plaques formed in fibroblast monolayers (Fig. 4), a phenotype that is consistent with Δ*ctaP* mutants that have a defect with adhesion (40). Using assays that demonstrated the cellular adhesion of beads covalently linked to purified CtaP, we previously showed that CtaP can function directly as an adhesin (40). L. monocytogenes strains lacking either permease were observed to be less efficient for host cell entry, but it remains to be determined whether this defect results from reduced binding of CtaP to the bacterial surface or if the permeases have a more direct role in adhesion. Peptide- and sugar-binding permeases of both known and unknown substrate specificities have been described to mediate adherence in different Streptococcus species (46, 52–56).

Schauer et al. also examined the roles of CtaP and its associated permeases in L. monocytogenes virulence and reported that a mutant containing a deletion of the entire transport system was slightly reduced in invasion and severely attenuated for intracellular replication but unaffected in adhesion to Caco-2 epithelial cells (57). Differences observed in adherence between their mutant and our mutants may reflect differences in cell lines used for *in vitro* studies and/or the genetic background of the parental strains (EGD versus 10403S). Nevertheless, both studies demonstrated the importance of the CtaP system to the pathogenesis of L. monocytogenes.

## MATERIALS AND METHODS

### Bacterial strains, plasmids, and growth conditions.

L. monocytogenes and Escherichia coli strains used in this study are listed in Table 1. L. monocytogenes 10403S (serotype 1/2a) is streptomycin resistant and was the parent strain used in the construction of all mutants. E. coli XL1-Blues (Agilent Technologies) and SM10 were used as host strains for maintenance and propagation of recombinant plasmids. L. monocytogenes and E. coli strains were grown at 37°C in BHI media (Difco Laboratories, Detroit, MI) and Luria broth (LB; Invitrogen Corp., Grand Island, NY), respectively. Bacteria containing the temperature-sensitive shuttle vector pKSV7 (58) or its recombinant plasmids were maintained using 50 μg/mL carbenicillin for E. coli and 10 μg/mL chloramphenicol for L. monocytogenes. Gram-negative/Gram-positive shuttle vector plasmid pPL2 (59), which integrates in single copy into a neutral site within the L. monocytogenes chromosome, was maintained using 25 μg/mL chloramphenicol for E. coli and 7.5 μg/mL chloramphenicol for L. monocytogenes. Kanamycin was used at a concentration of 50 μg/mL for maintenance of the L. monocytogenes integration plasmid pIMK2 (a kind gift of Colin Hill, University College Cork) in both E. coli and L. monocytogenes. Erythromycin selection of L. monocytogenes containing Em-marked deletions was carried out at a concentration of 1 μg/mL. Streptomycin 200 μg/mL was used in selection of L. monocytogenes following bacterial conjugation and for isolation of bacteria from organs of infected mice.

**TABLE 1:** Bacterial strains and plasmids used in this study

Strain	Description	Source or reference
*L.monocytogenes* strains		
NF-L100	Wild-type 10403S	57
NF-L1553	Δ*ctaP* (*lmo0135*)	40
NF-L1830	Δ*ctpP1* (*lmo0136*)	This study
NF-L1833	Δ*ctpP2* (*lmo0137*)	This study
NF-L1835	Δ*ctpP1* Δ*ctpP2* (*lmo0136/lmo0137*)	This study
NF-L1556	*inlA^m^* (InlA^S192N-Y369S^ mutant)	40
NF-L1558	Δ*ctaP*::*erm* mutation transduced with U153 phage library into *inlA^m^*	40
NF-L1958	Δ*ctpP1*::*erm* mutation transduced with U153 phage library into *inlA^m^*	This study
NF-L1961	Δ*ctpP2*::*erm* mutation transduced with U153 phage library into *inlA^m^*	This study
NF-L1964	Δ*ctP1* Δ*ctP2*::*erm* mutation transduced with U153 phage library into *inlA^m^*	This study
*E.coli* strains		
XL1 Blues	Propagation strain	Agilent Technologies
A Select	Propagation strain	Bioline
Plasmids		
pKSV7	Temperature- sensitive shuttle vector	59
NF-L1458	pKSV7 with *inl^m^* construct	40
NF-L1551	pKSV7 plus Δ*ctaP* deletion fragment with *erm* resistance cassette from pHY304 inserted in internal KpnI site	40
pNF-1792	pKSV7 plus Δ*ctpP1* deletion fragment with *erm* resistance cassette from pHY304 inserted in internal KpnI site	This study
pNF-1793	pKSV7 plus Δ*ctpP2* deletion fragment with *erm* resistance cassette from pHY304 inserted in internal KpnI site	This study
pNF-1795	pKSV7 plus Δ*ctpP1*Δ*ctpP2* deletion fragment with *erm* resistance cassette from pHY304 inserted in internal KpnI site	This study

### Plasmid and mutant strain constructions.

Primer pairs used for construction of in-frame deletions of *lmo0136* (*ctpP1*), *lmo0137* (*ctpP2*), and double deletion of *lmo0136* and *lmo0137* were generated by cloning 600 bp of the immediate upstream and downstream regions of their respective coding regions into the temperature-sensitive shuttle plasmid pKSV7 (58), leaving only the translational start and stop of the open reading frames. The flanking regions were amplified using splicing by overlap extension (SOEing) PCR (60). SOEing PCR product was digested with PstI and XbaI and ligated into the temperature-sensitive shuttle vector pKSV7 to generate the deletion construct. The *erm*(B) gene encoding erythromycin (Em) resistance and containing its native promoter was PCR amplified from pHY-304 (a kind gift from Craig Rubens and Amanda Jones) and inserted between the internal KpnI sites of the flanking regions in the pKSV7-based deletion constructs. The resulting plasmid constructs were transformed into the L. monocytogenes wild-type strain 10403S. Deletion mutants were selected based on Em^R^ and Cm^S^ and confirmed by PCR amplification of products from L. monocytogenes chromosomal DNA using the *em* gene primers in combination with primer pairs that are located outside the regions used for cloning.

The integration plasmid, pIMK2, was used for complementation of the various permease mutants. This plasmid is a derivative of the conjugative plasmid, pPL2, and integrates in single copy into the L. monocytogenes 10403S chromosome at a phage attachment site within the tRNA^Arg^ gene following conjugation (61). For construction of the permease complementation vectors, the entire open reading frame of each permease gene beginning with the second codon and ending with the stop codon was PCR amplified and digested with NcoI and PstI, subsequently ligated together with the pIMK2 vector, and transformed into E. coli XL1-Blues. The resulting construct was electroporated into the various permease deletion strains using a method previously described (61), and complemented strains were selected for kanamycin resistance.

Transduction of the permease mutations into different background strains was performed as previously described (62, 63). Briefly, high-titer U153 phage lysates were prepared from the individual permease mutants and mixed with mid-exponential-phase L. monocytogenes grown at 30°C in the presence of 10 mM MgSO_4_ and 10 mM CaCl_2_. The mixture was incubated at room temperature for 40 min, followed by plating on Em at 1 μg/mL BHI agar plates for selection of Δ*permease*::*erm* transductants. Plates were incubated at 37°C and Em-resistant transductants were isolated and confirmed by PCR.

### Amino acid *in vitro* growth assays.

HTM minimal medium was prepared as described elsewhere (64). Where indicated, specific amino acids (Sigma-Aldrich, St. Louis, MO) were added to the HTM media at concentrations of 0.1 or 0.3 mg/mL. For preparation of bacterial samples for HTM growth curves, overnight cultures of strains were grown in BHI at 37°C with shaking. Samples were normalized to optical density at 600 nm (OD_600_), pelleted, and washed twice in phosphate-buffered saline (PBS) before resuspension in PBS to a final volume equal to the normalized culture volume. Bacterial suspensions were diluted 1:20 in HTM, and growth in HTM media containing the various supplements was measured as the OD_600_ at indicated time points.

### Whole-genome shotgun microbial sequencing.

Total DNA was purified using Promega Maxwell RSC simply DNA cells kit, quantified using a Qubit fluorometer (Invitrogen), and analyzed for integrity using 2% agarose gel. Sequencing libraries were prepared using the Nextera DNA library preparation kit (Illumina catalog number PN 20018705) as per the manufacturer’s protocol. In brief, 50 ng of gDNA was used as a reaction input for each sample. The first DNA tagmentation step was followed by DNA cleanup and PCR amplification, using limited PCR cycles, to incorporate Illumina index adaptors. Next, individual libraries underwent double-sided bead purification, followed by pooling. The library pool was quantified using Qubit and analyzed for the fragment size distribution on TapeStation using high-sensitivity DNA tape (Agilent). Next, the library pool was sequenced on MiniSeq (Illumina) in order to check sequencing efficiencies and adjust accordingly the proportions of individual libraries. The concentration of the final adjusted library pool was confirmed by quantitative PCR using a KAPA library kit for Illumina (Roche). Sequencing was carried out on an Illumina NovaSeq 6000 instrument, S4 flow cell, with 2 × 150-bp paired-end reads, at the University of Illinois Roy J. Carver Biotechnology Center High-Throughput Sequencing and Genotyping Unit.

### L2 plaque assays.

Plaque assays were conducted as previously described (65). Briefly, murine L2 fibroblasts were grown to confluence in 6-well microtiter plates and infected with 20 μL of a normalized 1:20 dilution of overnight culture grown at 37°C in BHI with shaking (multiplicity of infection [MOI], 10:1). One hour postinfection, L2 infected monolayers were washed and 5 μg/mL of gentamicin was added to kill extracellular bacteria. Three days postinfection, neutral red (Sigma-Aldrich, St. Louis, MO) was added and plaques were visualized and measured using a micrometer (Finescale, Orange County, CA).

### Mouse infections.

**(i) Ethics statement.** All animal procedures were IACUC approved and performed under the strict guidelines of the BRL animal facilities at the University of Illinois at Chicago.

**(ii) Procedures.** For intravenous tail vein injections, *Listeria* strains were grown in BHI with the appropriate antibiotics with shaking at 37°C to an OD_600_ of ~0.6. Bacterial cells were collected by centrifugation, washed in PBS, and resuspended in PBS to a final concentration of 1 × 10^5^ CFU/mL. 8- to 10-week-old female Swiss Webster mice (Harlan Laboratories, Indianapolis, IN) were infected by tail vein injection with 0.2 mL of bacteria in PBS (2 × 10^4^ CFU). At 72 h postinfection, mice were sacrificed and the livers and spleens were harvested. Organs were placed in 5 mL of sterile water and tissues were homogenized using a Tissue Master 125 (Omni International, Marietta, GA). Tenfold serial dilutions were plated on BHI agar plates containing 200 μg/mL streptomycin (Sigma-Aldrich, St. Louis, MO) for enumeration of bacterial loads in each organ.

For intragastric infections, *Listeria* strains were grown in BHI with shaking at 37°C to an OD_600_ of ~0.6. Bacterial cells were collected by centrifugation, washed twice in PBS, and resuspended in PBS to a final concentration of 5 × 10^8^ CFU/mL. Groups of 8- to 10-week-old C57BL/6 mice (Charles River Laboratories) were orally inoculated with 0.2 mL of bacterial culture containing 1 × 10^8^ CFU of the various strains and 20 mg CaCO_3_ using an 18-gauge feeding tube (Solomon Scientific, Plymouth Meeting, PA) attached to a 1-mL syringe. Food was removed 24 h prior to infection. At 72 h postinfection, the mice were sacrificed and the stomach, intestine, liver, and spleen were harvested to determine bacterial CFU in these organs as described above.

### Statistical analyses.

Statistical analysis was performed using Prism software (GraphPad v.9.3.0). All multiple comparison analysis were compared to the wild-type control, unless otherwise stated. To find out whether there was a difference in the dependent variable for two independent groups, the Mann-Whitney test was used. The two-tailed unpaired Student's *t* test was used to identify statistically significant differences, where a *P* value of <0.05 was considered significant.

### Data availability.

Whole bacterial genome sequence data have been deposited in NCBI SRA under accession number PRJNA958163, release date 22 April 2023.

## References

[1] Radoshevich L, Cossart P. 2018. Listeria monocytogenes: towards a complete picture of its physiology and pathogenesis. Nat Rev Microbiol 16:32–46. doi:10.1038/nrmicro.2017.126. 29176582

[2] Schlech WF. 2019. Epidemiology and clinical manifestations of Listeria monocytogenes infection. Microbiol Spectr 7. doi:10.1128/microbiolspec.GPP3-0014-2018. PMC11026082 31837132

[3] Xayarath B, Freitag NE. 2012. Optimizing the balance between host and environmental survival skills: lessons learned from Listeria monocytogenes. Fut Microbiol 7:839–852. doi:10.2217/fmb.12.57. PMC3479242 22827306

[4] Gahan CG, Hill C. 2014. Listeria monocytogenes: survival and adaptation in the gastrointestinal tract. Front Cell Infect Microbiol 4:9. doi:10.3389/fcimb.2014.00009. 24551601 PMC3913888

[5] Chen GY, Pensinger DA, Sauer JD. 2017. Listeria monocytogenes cytosolic metabolism promotes replication, survival, and evasion of innate immunity. Cell Microbiol 19:e12762. doi:10.1111/cmi.12762. PMC5587384 28656691

[6] Bruno JC, Jr, Freitag NE. 2011. Listeria monocytogenes adapts to long-term stationary phase survival without compromising bacterial virulence. FEMS Microbiol Lett 323:171–179. doi:10.1111/j.1574-6968.2011.02373.x. 22092717 PMC3227008

[7] Rogalla D, Bomar PA. 2021. Listeria monocytogenes. StatPearls Publishing, Treasure Island, FL. https://www.ncbi.nlm.nih.gov/books/NBK430685. 30521259

[8] Jordan K, McAuliffe O. 2018. Listeria monocytogenes in foods. Adv Food Nutr Res 86:181–213. doi:10.1016/bs.afnr.2018.02.006. 30077222

[9] Poulsen KP, Czuprynski CJ. 2013. Pathogenesis of listeriosis during pregnancy. Anim Health Res Rev 14:30–39. doi:10.1017/S1466252312000242. 23347534

[10] Lamond NM, Freitag NE. 2018. Vertical transmission of Listeria monocytogenes: probing the balance between protection from pathogens and fetal tolerance. Pathogens 7:52. doi:10.3390/pathogens7020052. 29799503 PMC6027155

[11] Lamond NM, McMullen PD, Paramasvaran D, Visvahabrathy L, Eallonardo SJ, Maheswhari A, Freitag NE. 2021. Cardiotropic isolates of Listeria monocytogenes with enhanced vertical transmission dependent upon the bacterial surface protein InlB. Infect Immun 89:e00321-20. doi:10.1128/IAI.00321-20. 33139387 PMC7822136

[12] Jones GS, D'Orazio SE. 2013. Listeria monocytogenes: cultivation and laboratory maintenance. Curr Protoc Microbiol 31:9B.2.1–9B.2.7. 10.1002/9780471729259.mc09b02s31 PMC3920655 24510292

[13] Sauer J-D, Herskovits AA, O’Riordan MX. 2019. Metabolism of the Gram-Positive bacterial pathogen Listeria monocytogenes. Microbiol Spectr 7. doi:10.1128/microbiolspec.GPP3-0066-2019. PMC6699642 31418407

[14] O'Riordan M, Moors MA, Portnoy DA. 2003. Listeria intracellular growth and virulence require host-derived lipoic acid. Science 302:462–464. doi:10.1126/science.1088170. 14564012

[15] Keeney K, Colosi L, Weber W, O'Riordan M. 2009. Generation of branched-chain fatty acids through lipoate-dependent metabolism facilitates intracellular growth of Listeria monocytogenes. J Bacteriol 191:2187–2196. doi:10.1128/JB.01179-08. 19181817 PMC2655518

[16] Stoll R, Goebel W. 2010. The major PEP-phosphotransferase systems (PTSs) for glucose, mannose and cellobiose of Listeria monocytogenes, and their significance for extra- and intracellular growth. Microbiology (Reading) 156:1069–1083. doi:10.1099/mic.0.034934-0. 20056707

[17] Cao TN, Joyet P, Aké FMD, Milohanic E, Deutscher J. 2019. Studies of the Listeria monocytogenes cellobiose transport components and their impact on virulence gene repression. J Mol Microbiol Biotechnol 29:10–26. doi:10.1159/000500090. 31269503

[18] Keeney KM, Stuckey JA, O'Riordan MX. 2007. LplA1-dependent utilization of host lipoyl peptides enables Listeria cytosolic growth and virulence. Mol Microbiol 66:758–770. doi:10.1111/j.1365-2958.2007.05956.x. 17908209 PMC2367003

[19] Saier MH. Jr. 2015. The bacterial phosphotransferase system: new frontiers 50 years after its discovery. J Mol Microbiol Biotechnol 25:73–78. doi:10.1159/000381215. 26159069 PMC4512285

[20] Aboulwafa M, Zhang Z, Saier MH. Jr. 2019. Protein:protein interactions in the cytoplasmic membrane apparently influencing sugar transport and phosphorylation activities of the E. coli phosphotransferase system. PLoS One 14:e0219332. doi:10.1371/journal.pone.0219332. 31751341 PMC6872149

[21] Barabote RD, Saier MH, Jr. 2005. Comparative genomic analyses of the bacterial phosphotransferase system. Microbiol Mol Biol Rev 69:608–634. doi:10.1128/MMBR.69.4.608-634.2005. 16339738 PMC1306802

[22] Marquis H, Bouwer HG, Hinrichs DJ, Portnoy DA. 1993. Intracytoplasmic growth and virulence of Listeria monocytogenes auxotrophic mutants. Infect Immun 61:3756–3760. doi:10.1128/iai.61.9.3756-3760.1993. 8359896 PMC281074

[23] Borezee E, Pellegrini E, Berche P. 2000. OppA of Listeria monocytogenes, an oligopeptide-binding protein required for bacterial growth at low temperature and involved in intracellular survival. Infect Immun 68:7069–7077. doi:10.1128/IAI.68.12.7069-7077.2000. 11083832 PMC97817

[24] Verheul A, Hagting A, Amezaga MR, Booth IR, Rombouts FM, Abee T. 1995. A di- and tripeptide transport system can supply Listeria monocytogenes Scott A with amino acids essential for growth. Appl Environ Microbiol 61:226–233. doi:10.1128/aem.61.1.226-233.1995. 7887604 PMC167277

[25] Verheul A, Rombouts FM, Abee T. 1998. Utilization of oligopeptides by Listeria monocytogenes Scott A. Appl Environ Microbiol 64:1059–1065. doi:10.1128/AEM.64.3.1059-1065.1998. 9501445 PMC106367

[26] Wouters JA, Hain T, Darji A, Hüfner E, Wemekamp-Kamphuis H, Chakraborty T, Abee T. 2005. Identification and characterization of di- and tripeptide transporter DtpT of Listeria monocytogenes EGD-e. Appl Environ Microbiol 71:5771–5778. doi:10.1128/AEM.71.10.5771-5778.2005. 16204487 PMC1265990

[27] Berntsson RP-A, Doeven MK, Fusetti F, Duurkens RH, Sengupta D, Marrink S-J, Thunnissen A-MWH, Poolman B, Slotboom D-J. 2009. The structural basis for peptide selection by the transport receptor OppA. EMBO J 28:1332–1340. doi:10.1038/emboj.2009.65. 19300437 PMC2683046

[28] Segawa T, Johnson CM, Berntsson RP, Dunny GM. 2021. Two ABC transport systems carry out peptide uptake in Enterococcus faecalis: their roles in growth and in uptake of sex pheromones. Mol Microbiol 116:459–469. doi:10.1111/mmi.14725. 33817866 PMC8596753

[29] Wu TK, Wang YK, Chen YC, Feng JM, Liu YH, Wang TY. 2007. Identification of a Vibrio furnissii oligopeptide permease and characterization of its in vitro hemolytic activity. J Bacteriol 189:8215–8223. doi:10.1128/JB.01039-07. 17873048 PMC2168660

[30] Martin B, Prudhomme M, Alloing G, Granadel C, Claverys JP. 2000. Cross-regulation of competence pheromone production and export in the early control of transformation in Streptococcus pneumoniae. Mol Microbiol 38:867–878. doi:10.1046/j.1365-2958.2000.02187.x. 11115120

[31] Malone CL, Boles BR, Horswill AR. 2007. Biosynthesis of Staphylococcus aureus autoinducing peptides by using the synechocystis DnaB mini-intein. Appl Environ Microbiol 73:6036–6044. doi:10.1128/AEM.00912-07. 17693565 PMC2074992

[32] Domenech P, Kobayashi H, LeVier K, Walker GC, Barry CE. 3rd, 2009. BacA, an ABC transporter involved in maintenance of chronic murine infections with Mycobacterium tuberculosis. J Bacteriol 191:477–485. doi:10.1128/JB.01132-08. 18996991 PMC2620812

[33] Lyon GJ, Novick RP. 2004. Peptide signaling in Staphylococcus aureus and other Gram-positive bacteria. Peptides 25:1389–1403. doi:10.1016/j.peptides.2003.11.026. 15374643

[34] Goodell EW, Higgins CF. 1987. Uptake of cell wall peptides by Salmonella typhimurium and Escherichia coli. J Bacteriol 169:3861–3865. doi:10.1128/jb.169.8.3861-3865.1987. 3301822 PMC212484

[35] Alloing G, Martin B, Granadel C, Claverys JP. 1998. Development of competence in Streptococcus pneumonaie: pheromone autoinduction and control of quorum sensing by the oligopeptide permease. Mol Microbiol 29:75–83. doi:10.1046/j.1365-2958.1998.00904.x. 9701804

[36] Cundell DR, Weiser JN, Shen J, Young A, Tuomanen EI. 1995. Relationship between colonial morphology and adherence of Streptococcus pneumoniae. Infect Immun 63:757–761. doi:10.1128/iai.63.3.757-761.1995. 7868244 PMC173067

[37] Slamti L, Lereclus D. 2019. The oligopeptide ABC-importers are essential communication channels in Gram-positive bacteria. Res Microbiol 170:338–344. doi:10.1016/j.resmic.2019.07.004. 31376485

[38] Krypotou E, Scortti M, Grundström C, Oelker M, Luisi BF, Sauer-Eriksson AE, Vázquez-Boland J. 2019. Control of bacterial virulence through the peptide signature of the habitat. Cell Rep 26:1815–1827.e5. doi:10.1016/j.celrep.2019.01.073. 30759392 PMC6389498

[39] Chakravarty D, Sahukhal G, Arick M, II, Davis ML, Donaldson JR. 2021. Transcriptomic analysis of Listeria monocytogenes in response to bile under aerobic and anaerobic conditions. Front Microbiol 12:754748. doi:10.3389/fmicb.2021.754748. 34867878 PMC8636025

[40] Xayarath B, Marquis H, Port GC, Freitag NE. 2009. Listeria monocytogenes CtaP is a multifunctional cysteine transport-associated protein required for bacterial pathogenesis. Mol Microbiol 74:956–973. doi:10.1111/j.1365-2958.2009.06910.x. 19818015 PMC2802666

[41] Xayarath B, Alonzo F, III, Freitag NE. 2015. Identification of a peptide-pheromone that enhances Listeria monocytogenes escape from host cell vacuoles. PLoS Pathog 11:e1004707. doi:10.1371/journal.ppat.1004707. 25822753 PMC4379056

[42] Braibant M, Gilot P, Content J. 2000. The ATP binding cassette (ABC) transport systems of Mycobacterium tuberculosis. FEMS Microbiol Rev 24:449–467. doi:10.1111/j.1574-6976.2000.tb00550.x. 10978546

[43] Higgins CF, Linton KJ. 2004. The ATP switch model for ABC transporters. Nat Struct Mol Biol 11:918–926. doi:10.1038/nsmb836. 15452563

[44] Cundell DR, Tuomanen EI. 1994. Receptor specificity of adherence of Streptococcus pneumoniae to human type-II pneumocytes and vascular endothelial cells in vitro. Microb Pathog 17:361–374. doi:10.1006/mpat.1994.1082. 7752878

[45] Quentin Y, Fichant G, Denizot F. 1999. Inventory, assembly and analysis of Bacillus subtilis ABC transport systems. J Mol Biol 287:467–484. doi:10.1006/jmbi.1999.2624. 10092453

[46] Sun AN, Camilli A, Portnoy DA. 1990. Isolation of Listeria monocytogenes small-plaquemutants defective for intracellular growth and cell-to-cell spread. Infect Immun 58:3770–3778. doi:10.1128/iai.58.11.3770-3778.1990. 2172168 PMC313727

[47] Cundell DR, Pearce BJ, Sandros J, Naughton AM, Masure HR. 1995. Peptide permeases from Streptococcus pneumoniae affect adherence to eucaryotic cells. Infect Immun 63:2493–2498. doi:10.1128/iai.63.7.2493-2498.1995. 7790061 PMC173333

[48] Liu W, Huang L, Su Y, Qin Y, Zhao L, Yan Q. 2017. Contributions of the oligopeptide permeases in multistep of Vibrio alginolyticus pathogenesis. Microbiologyopen 6:e00511. doi:10.1002/mbo3.511. 28714216 PMC5635161

[49] Wollert T, Heinz DW, Schubert WD. 2007. Thermodynamically reengineering the listerial invasion complex InlA/E-cadherin. Proc Natl Acad Sci USA 104:13960–13965. doi:10.1073/pnas.0702199104. 17715295 PMC1955803

[50] Brenner M, Friedman S, Haber A, Livnat-Levanon N, Borovok I, Sigal N, Lewinson O, Herskovits AA. 2022. Listeria monocytogenes TcyKLMN cystine/cysteine transporter facilitates glutathione synthesis and virulence gene expression. mBio 13:e0044822. doi:10.1128/mbio.00448-22. 35435705 PMC9239247

[51] Loh E, Dussurget O, Gripenland J, Vaitkevicius K, Tiensuu T, Mandin P, Repoila F, Buchrieser C, Cossart P, Johansson J. 2009. A trans-acting riboswitch controls expression of the virulence regulator PrfA in Listeria monocytogenes. Cell 139:770–779. doi:10.1016/j.cell.2009.08.046. 19914169

[52] Jenkinson HF, Easingwood RA. 1990. Insertional inactivation of the gene encoding a 76-kilodalton cell surface polypeptide in Streptococcus gordonii Challis has a pleiotropic effect on cell surface composition and properties. Infect Immun 58:3689–3697. doi:10.1128/iai.58.11.3689-3697.1990. 2228239 PMC313715

[53] Jenkinson HF. 1992. Adherence, coaggregation, and hydrophobicity of Streptococcus gordonii associated with expression of cell surface lipoproteins. Infect Immun 60:1225–1228. doi:10.1128/iai.60.3.1225-1228.1992. 1339408 PMC257617

[54] Sutcliffe IC, Tao L, Ferretti JJ, Russell RR. 1993. MsmE, a lipoprotein involved in sugar transport in Streptococcus mutans. J Bacteriol 175:1853–1855. doi:10.1128/jb.175.6.1853-1855.1993. 8449892 PMC203995

[55] Russell RR, Aduse-Opoku J, Sutcliffe IC, Tao L, Ferretti JJ. 1992. A binding protein-dependent transport system in Streptococcus mutans responsible for multiple sugar metabolism. J Biol Chem 267:4631–4637. doi:10.1016/S0021-9258(18)42880-3. 1537846

[56] Kolenbrander PE, Andersen RN, Ganeshkumar N. 1994. Nucleotide sequence of the Streptococcus gordonii PK488 coaggregation adhesin gene, scaA, and ATP-binding cassette. Infect Immun 62:4469–4480. doi:10.1128/iai.62.10.4469-4480.1994. 7927711 PMC303132

[57] Schauer K, Geginat G, Liang C, Goebel W, Dandekar T, Fuchs TM. 2010. Deciphering the intracellular metabolism of Listeria monocytogenes by mutant screening and modelling. BMC Genomics 11:573. doi:10.1186/1471-2164-11-573. 20955543 PMC3091722

[58] Bishop DK, Hinrichs DJ. 1987. Adoptive transfer of immunity to Listeria monocytogenes. The influence of in vitro stimulation on lymphocyte subset requirements. J Immunol 139:2005–2009. doi:10.4049/jimmunol.139.6.2005. 3114382

[59] Smith K, Youngman P. 1992. Use of a new integrational vector to investigate compartment-specific expression of the Bacillus subtilis spoIIM gene. Biochimie 74:705–711. doi:10.1016/0300-9084(92)90143-3. 1391050

[60] Lauer P, Chow MY, Loessner MJ, Portnoy DA, Calendar R. 2002. Construction, characterization, and use of two Listeria monocytogenes site-specific phage integration vectors. J Bacteriol 184:4177–4186. doi:10.1128/JB.184.15.4177-4186.2002. 12107135 PMC135211

[61] Thornton JA. 2016. Splicing by overlap extension PCR to obtain hybrid DNA products. Methods Mol Biol 1373:43–49. doi:10.1007/7651_2014_182. 25646606

[62] Monk IR, Gahan CG, Hill C. 2008. Tools for functional postgenomic analysis of Listeria monocytogenes. Appl Environ Microbiol 74:3921–3934. doi:10.1128/AEM.00314-08. 18441118 PMC2446514

[63] Shetron-Rama LM, Mueller K, Bravo JM, Bouwer HG, Way SS, Freitag NE. 2003. Isolation of Listeria monocytogenes mutants with high-level in vitro expression of host cytosol-induced gene products. Mol Microbiol 48:1537–1551. doi:10.1046/j.1365-2958.2003.03534.x. 12791137

[64] Hodgson DA. 2000. Generalized transduction of serotype 1/2 and serotype 4b strains of Listeria monocytogenes. Mol Microbiol 35:312–323. doi:10.1046/j.1365-2958.2000.01643.x. 10652092

[65] Tsai HN, Hodgson DA. 2003. Development of a synthetic minimal medium for Listeria monocytogenes. Appl Environ Microbiol 69:6943–6945. doi:10.1128/AEM.69.11.6943-6945.2003. 14602660 PMC262277

