# Supplementary material for: Two Permeases Associated with the Multifunctional CtaP Cysteine Transport System in Listeria monocytogenes Play Distinct Roles in Pathogenesis
Source: Microbiol Spectr. 2023 May 18;11(3):e03317-22. doi: 10.1128/spectrum.03317-22 (PMC10269559; doi:10.1128/spectrum.03317-22)
Supplement: Supplemental file 1 — Supplemental material. Download spectrum.03317-22-s0001.pdf, PDF file, 0.1 MB [file spectrum.03317-22-s0001.pdf]

Supplemental Figure 1

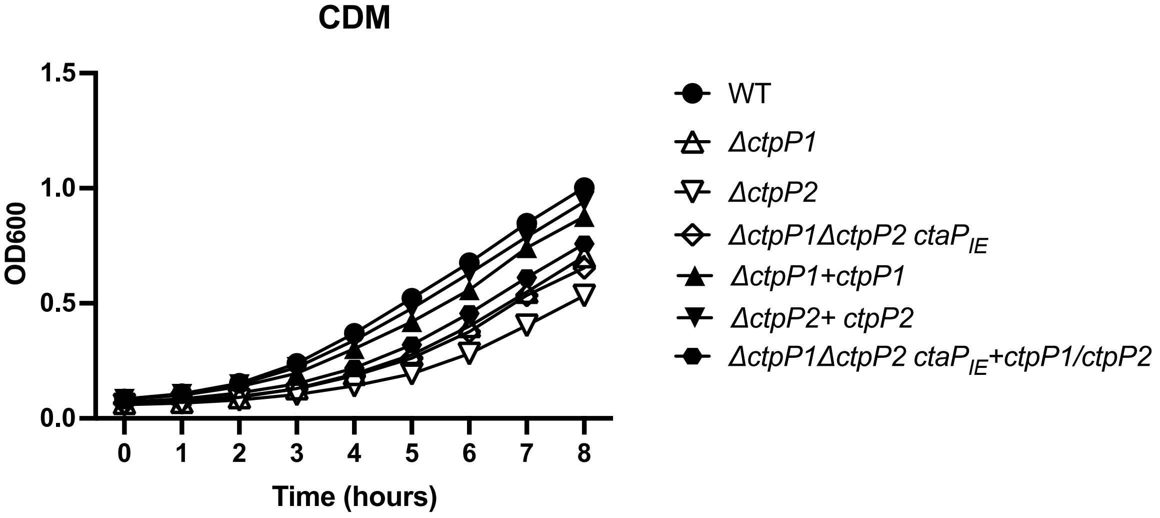

***ctpP1* and *ctpP2* exhibit differential growth patterns in chemically defined media.**

Assessment of growth of the permease mutants and complements in CDM minimal media. Overnight cultures of each strain grown shaking in CDM at 37°C were diluted 1:10 in fresh CDM media and the OD_{600} was measured at the indicated time points.

SUPPLEMENTAL METHODS

Chemically defined medium growth curve. Chemically defined medium (CDM) was prepared as previously described (50). Briefly, *Lm* strains were grown shaking in CDM overnight at 37°C and diluted 1:10 in fresh CDM; the OD_{600} measurements were taken at the indicated times.
